# C-754-02. Cellular Cross Talk in Early Burn Injury: Using Spatial Transcriptomics to Define the Burn Microenvironment

**DOI:** 10.1093/jbcr/irag033.030

**Published:** 2026-04-06

**Authors:** Angela Gibson, Mary Junak, Lingxin Cheng, Aiping Liu, Di Yan, Christina Kendziorski

**Affiliations:** University of Wisconsin Burn and Wound Center, Madison, WI; University of Wisconsin Burn and Wound Center, Madison, WI; University of Wisconsin School of Medicine and Public Health, Madison, WI; University of Wisconsin Burn and Wound Center, Madison, WI; University of Wisconsin School of Medicine and Public Health, Madison, WI; University of Wisconsin, Madison, WI

## Abstract

**Introduction:**

Spatial Transcriptomics (ST) represents a unique technology to identify gene expression in a tissue context dependent fashion in burn injury. Indeterminate burns have the potential to heal without surgery, however reversible injury and regenerative capacity is not well-defined. Using ST we sought to describe the cellular makeup, gene expression profile and cell–cell communication in the early phase after burn injury.

**Methods:**

Tissue biopsies were collected from indeterminate depth burns in 4 adult patients (age 18-62; TBSA 7-25%) on post burn day 3 or 4. Samples were formalin-fixed and paraffin-embedded. Hematoxylin and eosin staining (H&E) was performed to evaluate tissue architecture. Visium HD whole genome spatial transcriptome pipeline workflow was performed on tissue sections. Data underwent cell segmentation by bin2cell to convert 2x2 μm bins to cells, and quality control was performed to filter out low transcript counts. The filtered data was clustered with Seurat Louvain method and annotation was performed on the clusters using CASSIA. Genes of interest were selected to infer cell–cell communications using CellChat and current literature of ligand-receptor databases.

**Results:**

Three out of the 4 patients underwent excision and grafting. At the time of biopsy, burn depth ratio of nonviable to viable cells was 0.16-0.30 of total tissue depth. Analyses of gene expression coregistered with the H&E of the tissue biopsy revealed multiple cell types present throughout the tissues in the expected locations. Neutrophils predominately clustered in the superficial damaged region of the tissue in all samples with gene expression reflecting inflammation such as CXCL8, TIMP1, S100A8, S100A9, and C5AR1. In all samples, we identified ligand-receptor colocalization throughout the tissues, most abundantly in the 1 patient sample that was allowed to heal without surgery. Notably, robust colocalization of ligand-receptor pairs TIMP1-CD63, TIMP-LRP1, S100A9- ITGB2, MMP-LRP1 and MMP9-CD44 were identified throughout the entire tissue in all 4 samples, others (CXCL8-CXCR1, CXCLK8-CXCR2, CXCL8-ACKR1) were localized at the superficial aspect of the tissues.

**Conclusions:**

Gene expression and ligand-receptor colocalization in early indeterminate depth burn tissue is associated with cell survival, migration, proliferation, angiogenesis, and ECM remodeling. ST is promising as a powerful tool to define the cellular landscape and function in indeterminate depth burn injury, although exploration is in its infancy. This technology has the potential to elucidate key mechanisms underlying burn wound progression and facilitate development of potential therapeutic targets to reduce or eliminate progression of injury.

**Applicability of Research to Practice:**

Understanding the microenvironment at the early stages of human burn injury may allow the development of treatments that reduce or eliminate the need for skin grafting and improve healing outcomes.

**Funding for the study:**

NIGMS R01GM145723.

